# Chemoinformatics in drug development

**DOI:** 10.1186/1758-2946-4-S1-O5

**Published:** 2012-05-01

**Authors:** Colin R Groom

**Affiliations:** 1 Cambridge Crystallographic Data Centre, 12 Union Road, Cambridge CB2 1EZ, UK

## 

It would be unimaginable to prosecute a drug discovery program without applying appropriate chemoinformatics analyses. In recent years a focus on target affinity and activity has been complemented by techniques to address physico-chemical properties such as lipophilicity and solubility, biological properties such as absorption, distribution, metabolism, elimination and toxicity. As such, a rounded package of studies can be performed to help in the generation and selection of molecules as clinical development candidates. Medicinal chemists are often well-served by their computational chemistry colleagues. Not so the development chemist.

Having successfully produced a clinical candidate the attention of chemoinformaticians in the pharmaceutical industry usually turns to the next molecule and scant regard is given to the contributions that can be made as a candidate molecule progresses towards becoming part of a drug substance.

This presentation will highlight the opportunities for the application of chemoinformatics techniques to the area of pharmaceutical materials science, a critical and complex phase in the creation of a drug.

